# Ocular mycobacterial lesions in cats

**DOI:** 10.1177/03009858221098431

**Published:** 2022-05-19

**Authors:** Jordan L. Mitchell, Laura MacDougall, Melanie J. Dobromylskyj, Ken Smith, Renata Stavinohova, Danièlle A. Gunn-Moore, Jayne C. Hope, Emma Scurrell

**Affiliations:** 1 The University of Edinburgh, Midlothian, UK; 2 The Wheelhouse Veterinary Centre, Chesham, UK; 3 Finn Pathologists, Diss, UK; 4 Royal Veterinary College, Hatfield, UK; 5 Lumbry Park Veterinary Specialists, Alton, UK; 6 Cytopath Ltd, Ledbury, UK

**Keywords:** cats, eye, mycobacteria, tuberculosis, histopathology, immunohistochemistry

## Abstract

Ocular mycobacterial infections are an under-recognized cause of morbidity in the domestic cat. This study aimed to explore the distribution, histopathological appearance, and severity of feline ocular mycobacterial lesions, and to characterize the immune cell population with immunohistochemistry. Routine histological staining with hematoxylin and eosin, and Masson’s trichrome, was performed to identify ocular lesions and assign an inflammation score based on the number of cells present. Acid-fast bacilli were detected with Ziehl-Neelsen, and immunohistochemistry for ionized calcium-binding adaptor protein-1 (Iba1), calprotectin, cluster of differentiation 3 (CD3), and Pax5 was undertaken on formalin-fixed paraffin-embedded tissue samples from 24 cases of ocular mycobacteriosis. Posterior or panuveitis with concurrent retinitis was identified in 20/24 cases (83%), with retinal detachment in 16/20 (80%) of these cases. Choroidal lesions had the highest median inflammation score. Ziehl-Neelsen-positive organisms were detected in 20/24 cases (83%), with the highest prevalence of acid-fast bacilli detected in choroidal lesions (16/20, 80%). Lesions were typically granulomatous to pyogranulomatous, characterized by abundant numbers of Iba1-positive macrophages, followed by calprotectin-positive granulocytes and monocytes, fewer T cells, and rarer B cells. However, where iritis was identified, inflammation was typically lymphoplasmacytic (11/16 cases, 69%). Where diagnostic testing was performed, tuberculosis (ie, infection with *Mycobacterium bovis*, *Mycobacterium microti*, or a nonspeciated *Mycobacterium tuberculosis*-complex pathogen) was diagnosed in 20/22 cats (91%), with *Mycobacterium lepraemurium* infection identified in the other 2/22 cats (9%). These results suggest the choroid is the primary site of lesion development in most cases of feline ocular mycobacteriosis, and inflammatory changes are associated with the presence of mycobacteria localized to ocular tissues.

Feline mycobacterial disease is increasingly recognized in Great Britain, and the mycobacteria most commonly identified on culture are those that cause tuberculosis (TB), namely, infection with members of the *Mycobacterium tuberculosis*-complex (MTBC), including *Mycobacterium bovis* and *Mycobacterium microti*.^34^ Regardless of the specific cause, clinical lesions can appear grossly similar.^32^ Typically, cases present with cutaneous lesions, which may or may not be ulcerated, and sinus tracts with draining purulent discharge may be present; local lymphadenopathy may also be reported.^34^ Less common presentations of feline mycobacterial disease include ocular,^82^ articular,^45^ and alimentary TB, the last of which was recently reported as an outbreak associated with feeding a commercially available raw food diet.^62^

Ocular disease is reported in nearly 7% of domestic cats attending primary veterinary care in England,^64^ where it may be part of a systemic disease process or localized to the ocular, periocular, and orbital structures. Infectious feline ophthalmic diseases include toxoplasmosis,^14^ feline infectious peritonitis (FIP),^40,67^ and mycobacteriosis, among many others.^7^ Mycobacteria as a cause of ocular disease is less well appreciated, despite historical reports.^49^ A recent study showed that approximately 6% of cats with mycobacteriosis presented with ocular lesions,^34^ which could be seen in association with systemic disease.^82^ Clinical signs associated with feline ocular mycobacteriosis vary depending on the tissue(s) affected and route of infection, ranging from corneal or conjunctival granulomas,^18,46,59^ to uveitis,^82^ and even acute-onset blindness.^23,82^ Both MTBC and nontuberculous mycobacteria can cause ocular disease in cats,^16,18,19,23,25,31,46,54,59,82^ and treatment typically consists of a combination of surgery (ie, enucleation) and systemic antimycobacterial therapy, with remission rates of 80% having been reported.^82^

Diagnosing cases of feline mycobacteriosis is difficult, and histopathology is typically the first step in the process^35^; this is especially true for cases of ocular mycobacteriosis, where the eye is often enucleated as part of the diagnosis and the potential treatment. Granulomatous to pyogranulomatous inflammation dominated by epithelioid macrophages is the hallmark histopathological finding of feline mycobacterial lesions,^35,41^ with mineralization and multinucleated giant cells being less common. Ziehl-Neelsen (ZN) staining for acid-fast bacilli (AFB) morphologically consistent with mycobacteria is often negative, with only one-third of biopsy samples examined deemed to be ZN positive.^33^ The histopathological features of feline ocular mycobacteriosis have been described: most cases are associated with a granulomatous to pyogranulomatous chorioretinitis, with or without other histological lesions including retinal detachment.^23,82^

This study aimed to identify the tissues most frequently affected in cases of feline ocular mycobacteriosis, describe the inflammatory changes present, and thereby suggest the potential route of infection. The histopathological appearance and degree of inflammation were compared between affected tissues, and ZN staining was undertaken to quantify the load and location of AFB. Immunohistochemistry (IHC) for a range of immune cell markers was undertaken to further characterize the cellular composition of these lesions.

## Materials and Methods

### Animals and Samples

Ethical approval for this study was granted by the University of Edinburgh Veterinary Ethical Review Committee (Approval No. 79 14).

Formalin-fixed paraffin-embedded globes with a morphological diagnosis consistent with ocular or periocular mycobacteriosis (ie, a granulomatous or pyogranulomatous inflammatory infiltrate, predominantly comprised of epithelioid macrophages, with or without necrosis) were provided by board certified pathologists at Cytopath, Herefordshire, UK and Finn Pathologists, Norfolk, UK. Additional tissues were held at the Roslin Institute at the University of Edinburgh, Scotland. The globes had been previously enucleated by licensed veterinarians as part of antemortem diagnostic investigation and management of ocular disease, or they were taken at postmortem examination. Where available, clinical records were provided following owner consent, and data were stored securely in accordance with data protection guidelines.

Samples were included in the study if they showed well-preserved ocular structures and had histopathological evidence of granulomatous to pyogranulomatous lesions typical of mycobacterial disease. ZN-negative samples were included if there was additional supporting evidence for mycobacterial disease, namely, demonstration of AFB in nonocular mycobacterial lesions from the same cat, a positive result on specialized mycobacterial culture, polymerase chain reaction (PCR) or interferon-gamma release assay (IGRA),^71^ or if the board certified pathologist considered the histopathological appearance of the lesion(s) inconsistent with that seen with FIP or other causes of ocular (pyo)granulomatous inflammation. Where available, signalment data, the results of feline leukemia virus antigen and feline immunodeficiency virus antibody testing, and other clinical examination findings were recorded.

Control tissues for IHC were a cutaneous lesion from a cat diagnosed with MTBC infection positive for calprotectin by IHC,^55^ the parietal lobe from a cat with cognitive dysfunction, positive for ionized calcium-binding adaptor protein-1 (Iba1), and a feline cutaneous nonepitheliotropic B-cell lymphoma with sparse non-neoplastic T cells. Calprotectin is expressed by granulocytes, monocytes, and recently blood-derived macrophages and granulocytes^74^; Iba1 by macrophages and microglia^39^; cluster of differentiation 3 (CD3) by T cells^41^; and Pax5 by B cells other than plasma cells.^2^

### Histopathology

Histopathology was performed to identify which ocular tissues contained inflammatory lesions, to describe the nature of the inflammatory cell population and score the intensity of inflammation based on the number of cells present. Four-micron thick sections were cut, mounted, and stained with hematoxylin and eosin (HE), ZN, and Masson’s trichrome (MT). Slides were scanned using a NanoZoomer-XR scanner, using the NanoZoomer Digital Pathology (NDP) sofware, NDP.scan Ver.3.2.12 (Hamamatsu Photonics) and images viewed on NDP.view2 Ver 2.7.52 (Hamamatsu Photonics, Hamamatsu City, Japan). Sections stained with HE were examined for the presence and population of inflammatory cells, the granuloma type (ie, “organized,” having singular or multifocal zones of central caseous necrosis surrounded by macrophages and neutrophils, with peripherally located lymphocytes and plasma cells; or “atypical,” having well-defined clusters of macrophages and epithelioid macrophages divided by thin fibrous septa),^55^ and additional features such as necrosis. The presence of collagen, indicating fibrosis, was assessed using the MT-stained slides. The inflammatory cell population was visually assessed and categorized as either: (1) (pyo)granulomatous if the dominant inflammatory phenotype consisted of macrophages and epithelioid macrophages, with variable numbers of neutrophils and fewer lymphocytes or plasma cells; (2) lymphoplasmacytic; or (3) mixed if both (pyo)granulomatous and lymphoplasmacytic cells were present in similar numbers, or if the pattern of inflammatory changes identified segmental regions of infiltration with either cell population within the same tissue. The degree of inflammation was scored by counting the number of inflammatory cells present within each lesion using the cell detection feature in QuPath Ver 0.1.2^8^ and assigned an inflammation score (Table 1). Slides stained with ZN were examined under light microscopy and the bacterial index for tissues with inflammatory lesions was calculated as previously described,^55^ and a bacterial index grade assigned (Table 2).

**Table 1.** Inflammation scoring system used to assign grades for the number of inflammatory cells present within ocular lesions.

Inflammation score	No. inflammatory cells
0	0
1 (minimal)	Less than 1000
2 (mild)	1000–10,000
3 (moderate)	10,001–50,000
4 (marked)	50,001–100,000
5 (extensive)	More than 100,000

**Table 2.** Bacterial index grading system used to describe the average number of AFB present over 15 hpfs, adapted from the Ridley scoring system used for cases of leprosy in humans.

Bacterial index grade	Bacterial index classification	Average number of AFB/hpf
0	Low	0
1	Low	0.01–0.1
2	Low	0.1–1
3	High	1–10
4	High	10–100
5	High	100–1000
6	High	Over 1000

Abbreviations: AFB, acid-fast bacilli; hpf, high-power field.

### Immunohistochemistry

Expression of calprotectin, Iba1, CD3, and Pax5 was detected using IHC. Briefly, sections were mounted on SuperFrost Plus-coated slides, dewaxed, rehydrated, rinsed in distilled water, and washed in Tris-buffered saline with Tween®20. All subsequent wash steps, and antibody dilutions, were in Tris-buffered saline with Tween®20. Antigen retrieval followed by incubation with primary antibodies for 30 minutes at room temperature (RT) was performed as per Table 3. To block for nonspecific endogenous peroxidase activity, REAL Peroxidase-Blocking Solution (Dako, Glostrup, Denmark) was used for 10 minutes at RT, before incubating sections with goat antimouse/antirabbit secondary detection polymer (EnVision™+ Dual Link System-HRP, Dako) for 40 minutes at RT. Positive labeling was visualized using Dako Liquid DAB+ Substrate Chromogen System for 10 minutes at RT. Slides were counterstained with hematoxylin and Scott’s Tap Water for 10 seconds each, dehydrated, cleared in xylene, mounted, and a cover-slip applied.

**Table 3.** Summary of the primary antibodies used and antigen retrieval methods for immunohistochemical investigation of the cell populations present in feline ocular tissues with mycobacterial lesions.

Target	Antibody	Source	Antigen retrieval	Primary antibody concentration or dilution
Calprotectin	Mouse monoclonal antihuman macrophages, clone MAC387 (MCA874G)	BioRad	Proteinase K (Dako) 20 minutes at RT	1:800 (1.25 µg/ml)
Iba1	Rabbit polyclonal anti-Iba1 (019-19741)	Wako	0.01 M sodium citrate buffer, pH 6.0 Overnight at 60°C	1:500 (1 µg/ml)
CD3	Rabbit polyclonal antihuman CD3 (A0452)	Dako	Proteinase K (Dako) 20 minutes at RT	1:50 (8 µg/ml)
Pax5	Mouse monoclonal antihuman B-cell-specific activator protein, clone DAK-Pax5 (M7307)	Dako	0.01 M sodium citrate buffer, pH 6.0 20 minutes at 121°C^a^	1:50 (3.14 µg/ml)

Abbreviation: RT, room temperature.

a Antigen retrieval performed using the Antigen Retriever 2100 (Aptum Biologics Ltd, UK).

A concentration-matched isotype control was used to assess nonspecific labeling (mouse IgG1 antibody, MCA928, BioRad, Hercules, CA, USA). Negative controls were run with omission of the primary antibody. Immunolabeled slides were scanned as previously described and examined to determine the relative degree of positive labeling for each IHC marker and the distribution of positive cells within the lesion(s).

## Results

### Study Population Characteristics

Twenty-six globes with a morphological diagnosis of mycobacteriosis were identified from archives and assessed for eligibility. Two globes were excluded due to lack of preservation of globe morphology and loss of definition of ocular structures, resulting in a final study population of 24 globes from 24 cats. Summarized details are available in Supplemental Table S1. The median age of cats with ocular mycobacteriosis was 6 years (range: 1 year–12 years, 6 months). There were slightly more male cats included in this study (13 male, 11 female); the neuter status was known for 19 individuals (male neutered, n = 11; female neutered, n = 8). The most common breed was Domestic Short- or Long-hair (n = 18); there were 3 British Shorthair, 1 Bengal and 1 Burmese, respectively, and the breed was not recorded for 1 animal. Ten of the cases presented in this study were described by Stavinohova et al^82^ (Supplemental Table S1), including clinical presentation, treatment, and outcomes.

Results of specialized culture, PCR, or IGRA were available for 22 cats; a diagnosis of TB (ie, infection with a member of the MTBC) was made in 20/22 cats (91%; cases 1–20). Mycobacterial culture was performed in 3 cases and was positive for *M. bovis* in 1 cat (case 1), and *M. microti* infection in 2 cats (cases 2 and 3). These 3 cats also underwent testing by IGRA, and the results correlated with the culture-confirmed diagnosis.^72^ Six cats were diagnosed with *M. bovis* infection by PCR (Genotype Mycobacterium and GenoType MTBC kits, Hain Lifescience GmbH, Nehren, Germany)^42,73^ (cases 4–9), while 7 further cats had a positive PCR result confirming MTBC infection, but further testing to identify the mycobacterial species was not performed either due to insufficient DNA, financial restrictions, or lack of test availability (cases 10–16). A concurrent IGRA was performed in 3 of these cats which indicated infection with *M. bovis* in 1 cat (case 10), and MTBC infection in the other 2 cats (cases 11 and 12). A further 4 cats were diagnosed by IGRA, with *M. bovis* infection suggested in 3 cats (cases 17–19) and MTBC infection in the remaining individual (case 20); this cat had consumed the commercial raw food associated with an outbreak of *M. bovis* TB.^63^ A diagnosis of *M. lepraemurium* was made in 2/22 cats (9%) by PCR and sequencing of the 16S rRNA product (cases 21 and 22). Culture, PCR, or IGRA was not performed for the remaining 2 cats (cases 23 and 24); therefore, a species-level diagnosis of mycobacterial infection was not attained for these 2 cases. However, case 23 was previously reported as having rare AFB,^82^ and case 24 had TB-specific antibodies demonstrated by an in-house enzyme-linked immunosorbent assay (data not shown), hence their inclusion in this study. Results of feline leukemia virus antigen and feline immunodeficiency virus antibody testing were available for 4 cats, and all were negative (cases 1, 10, 17, and 19).

### Lesion Distribution and Inflammation Score

**Figure 1.**
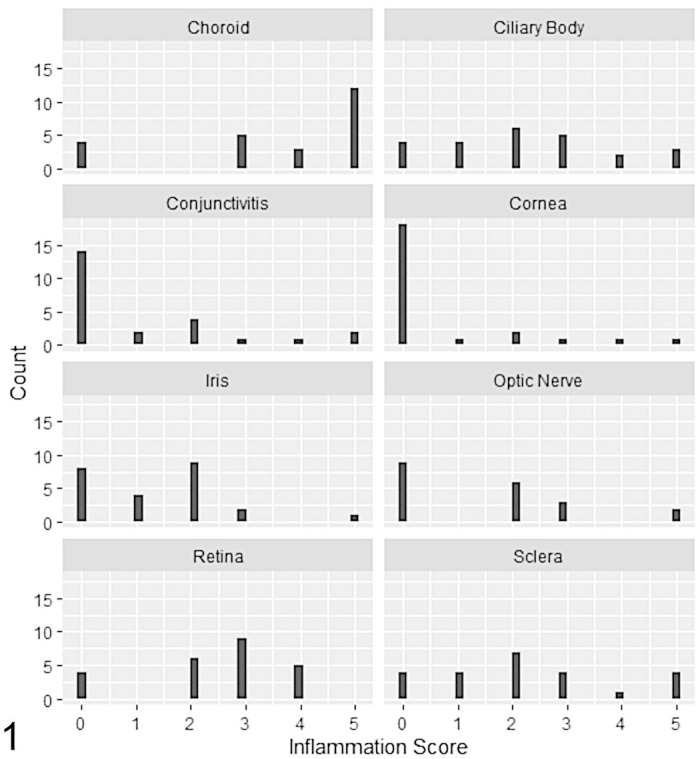
The histogram shows the inflammation score and location of ocular lesions identified in 24 cases of feline mycobacteriosis.

Histopathological examination of HE-stained sections identified changes consistent with mycobacterial infection, that is, granulomatous to pyogranulomatous inflammation with epithelioid macrophages, with or without necrosis, or infiltration with mixed inflammatory cells present across a range of ocular tissues, with varying degrees of involvement and severity within and between individuals (Supplemental Table S2). Inflammatory cells were identified most often within the choroid, retina, ciliary body, and sclera (20/24 cats, 83%). There was wide variation in inflammation scores across tissues (Fig. 1); where inflammation was present, the highest median inflammation score was recorded for choroidal lesions (median score = 5). All cats with choroidal lesions showed concurrent retinitis, although the degree of inflammation was less pronounced (median score = 3). Posterior uveitis or panuveitis with retinitis was the most common histological finding, recorded in 20/24 cats (83%; cases 1, 2, 4–14, 16–20, 23, and 24; Table 4). An appreciable inflammatory cell component was present within the vitreous cavity in 3 of these cats (cases 8, 17, and 18), resulting in an endophthalmitis. Anterior uveitis without choroidal involvement was identified in 1 cat (case 15). Histopathological evidence of optic neuritis was seen in 11/20 cats (55%; cases 1, 6–9, 12–14, 20, 23, and 24); the optic nerve was not present for evaluation in 4 cats (cases 4, 5, 21, and 22). Anterior mass lesions affecting the cornea, bulbar or palpebral conjunctiva, and/or sclera were recorded in 5/24 cats (21%; cases 2, 3, 16, 21, and 22); inflammation restricted to these tissues with no intraocular involvement was present in 3 of the 5 cats. In the 2 cats with *M. lepraemurium* infection, lesions were restricted to the cornea and sclera (case 21), and in 1 cat there was also infiltration of the bulbar conjunctiva with inflammatory cells (case 22). A conjunctival mass was the only histopathological finding in 1 cat diagnosed with *M. microti* (case 3), whereas posterior or panuveitis was recorded in the remaining cats that had a diagnosis of MTBC infection.

**Table 4.** Summary of the main histological findings in 24 cases of feline ocular mycobacteriosis.

Histological finding	No. cases	%	Case
Posterior uveitis or panuveitis with retinitis	20	83	1, 2, 4–14, 16–20, 23, 24
Optic neuritis	11	55^a^	1, 6–9, 12–14, 20, 23, 24
Corneal, conjunctival, and/or scleral mass	5	21	2, 3, 16, 21, 22
Orbital to periorbital cellulitis or abscess	3	13	4, 9, 12
Anterior uveitis	1	4	15

a Optic nerve not present in hematoxylin and eosin–stained sections in 4 cases for evaluation.

### Acid-Fast Bacilli

**Figure 2.**
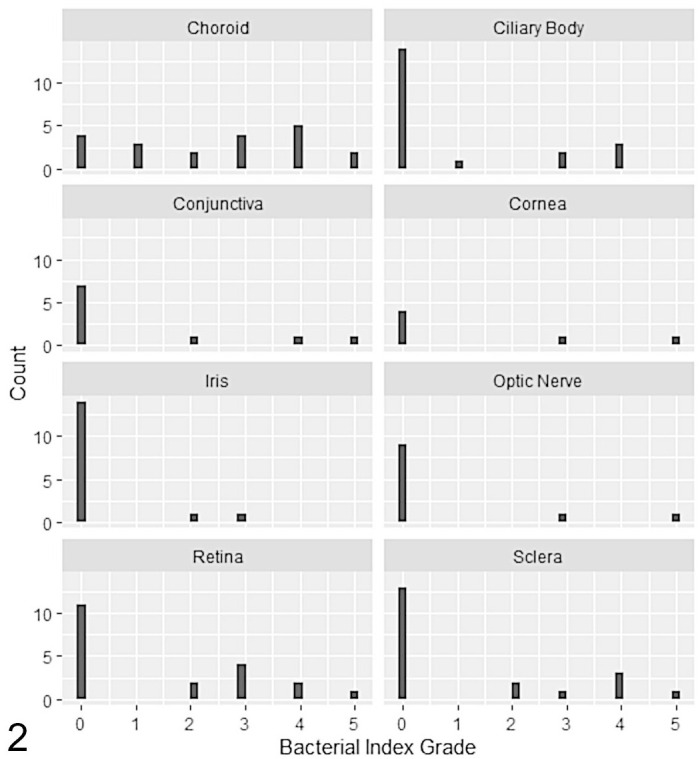
The histogram shows the bacterial index grade for inflammatory lesions split by tissue in 24 cases of feline ocular mycobacteriosis.

ZN staining demonstrated the presence of AFB morphologically consistent with mycobacteria in 20/24 globes (83%; cases 1–14, 16–21; Supplemental Table S2), although in 2 cats only 1 AFB was identified across all affected tissues (cases 19 and 20). AFB were identified in 16/20 (80%) choroidal lesions (cases 1, 2, 4–14, 17, 19, and 20); the median bacterial index grade for choroidal lesions was 3, compared with 0 for the other tissues examined, although there was a wide range of bacterial index grades observed across tissues (Fig. 2). Of the 5 cats with a mass lesion present in the anterior segment of the globe (cornea, bulbar, or palpebral conjunctiva and anterior sclera), AFB were detected in 4 of these (cases 2, 3, 16, and 21), with at least one of the affected tissues per case scoring with a high bacterial index grade (grade 3 or above). AFB were only observed in regions of (pyo)granulomatous inflammation, and extracellular AFB were frequently identified in regions of necrosis, sometimes in very large numbers.

### Choroid and Retina

**Figures 3–10.**
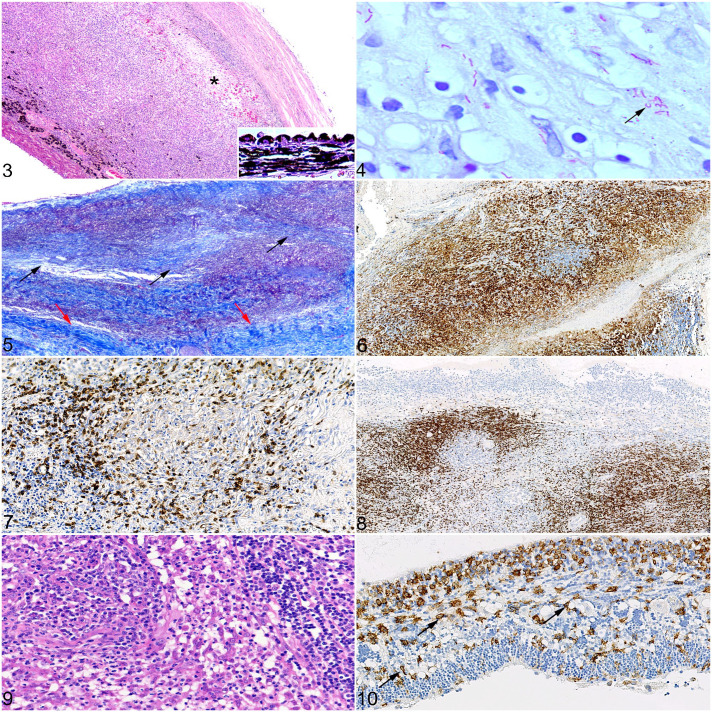
Mycobacteriosis, eye, cat. Inflammation scores: 1 = less than 1000 inflammatory cells; 2 = 1000–10,000 inflammatory cells; 3 = 10,001–50,000 inflammatory cells; 4 = 50,001–100,000 inflammatory cells; 5 = more than 100,000 inflammatory cells, calculated over the entire area of the lesion. **Figure 3.** Choroid, *Mycobacterium bovis*, case 7. Pyogranulomatous choroiditis, inflammation score 5. There is full-thickness infiltration of the choroid with inflammatory cells. Necrosis and edema are also present (asterisk). There is hypertrophy of the retinal pigment epithelium, indicating pathological retinal detachment (inset). Hematoxylin and eosin (HE). **Figure 4.** Choroid, *Mycobacterium microti*, case 2. Pyogranulomatous choroiditis, inflammation score 4, with evidence of acid-fast bacilli, some of which display the S-shaped morphology suggestive of *M. microti* (black arrow). Bacterial index grade 4. Ziehl-Neelsen (ZN). **Figure 5.** Choroid, *M. bovis*, case 1. Choroidal fibrosis is demonstrated by the presence of collagen fibers within the choroid (black arrows). Note the more densely arranged collagen fibers of the sclera (red arrows) compared with the collagen fibers within the choroid. Inflammation score 5. Masson’s trichrome. **Figure 6.** Choroid, *Mycobacterium tuberculosis*-complex (MTBC), case 13. Pyogranulomatous choroiditis, inflammation score 5, demonstrating abundant positive immunohistochemical labeling for ionized calcium-binding adaptor protein-1 (Iba1) on epithelioid macrophages, and lack of positivity on neutrophils and lymphocytes. Immunohistochemistry (IHC) for Iba1. **Figure 7.** Choroid, *M. bovis*, case 4. Inflammation score 5. Positive membranous labeling for T cells, forming the lymphocytic cuff in a granuloma. IHC for CD3. **Figure 8.** Choroid, *M. bovis*, case 17. Inflammation score 5. Large numbers of B cells infiltrating the choroid in a case of mixed pyogranulomatous and lymphoplasmacytic choroiditis. IHC for Pax5. **Figure 9.** Retina, MTBC, case 13. Macrophages and neutrophils are observed within the retina, with a lesser lymphoplasmacytic infiltrate. HE. **Figure 10.** Retina, *M. bovis*, case 18. Mixed pyogranulomatous and lymphoplasmacytic retinitis, inflammation score 3. Iba1-positive cells present within the inner layers of the retina, some of which have a more spindle-shaped morphology suggestive of resident microglia (black arrows). IHC for Iba1.

Choroidal lesions were recorded in 20/24 cases (83%; Fig. 3), and of these AFB were identified in 16/20 cases (80%; Fig. 4). The median inflammation score for choroidal lesions was higher than that for lesions in any other ocular tissue (median score = 5). The inflammatory population was ascribed as mixed in 11 cats (cases 2, 4, 5, 10–12, 14, 17, 20, 23, and 24), whereas the remaining 9 cats showed predominantly (pyo)granulomatous inflammation (cases 1, 6–9, 13, 16, 18, and 19). Granulomatous/pyogranulomatous inflammation was present across all layers of the choroid, but this often became restricted to the inner choroid in regions approaching the pars plana. This is in contrast to the lymphoplasmacytic infiltrate, which was mostly restricted to perivascular aggregates around the large and medium vessels in the outer choroid, although 5 cats did show segmental lymphoplasmacytic infiltration within regions of (pyo)granulomatous inflammation (cases 1, 2, 11, 20, and 24). “Organized” (pyo)granulomas were present in 9 cats (cases 4, 5, 7, 9, 10, 16, 19, 20, and 24), some of which were surrounded by an outer layer of concentric spindle-shaped cells, but encapsulating fibrosis was often minimal; there was 1 cat with “atypical” granulomas and abundant collagen deposition. There was no clear formation of (pyo)granulomas in 10 cats (cases 1, 2, 6, 8, 11, 13, 14, 17, 18, and 23); rather, the infiltrate formed a diffuse sheet of inflammatory cells, from herein termed “unstructured.” Multifocal zones of necrosis within regions of (pyo)granulomatous inflammation (“organized” or “atypical” (pyo)granulomas and unstructured sheets of inflammatory cells) were present in 17/20 cats (85%; cases 1, 4–13, 16–20, and 24); concurrent degenerative change (eg, vacuolation and necrosis of the tapetum lucidum) was also identified. Diffuse or segmental choroidal fibrosis was identified in 12/20 cats (60%; cases 1, 4–6, 9, 10, 14, 16, 17, 19, 20, and 24; Fig. 5), especially of the choriocapillaris layer while sparing the larger choroidal vessels.

On IHC, the most abundant cell types were Iba1-positive macrophages and epithelioid macrophages (Fig. 6). The intensity of labeling was greater for macrophages at the periphery of (pyo)granulomas, where present, with less intense labeling in macrophages at the center of the (pyo)granuloma. Calprotectin-positive monocytes and granulocytes were the second most abundant cell population present, often scattered throughout the (pyo)granulomatous inflammation and surrounding regions of necrosis. T cells were the next most common population, often forming peripheral cuffs particularly around “organized” (pyo)granulomas (Fig. 7), or diffusely scattered throughout “unstructured” (pyo)granulomatous lesions. In contrast, B cells were rarely associated with (pyo)granulomas or “unstructured” (pyo)granulomatous inflammation; these were the predominant cell type in regions of segmental lymphoplasmacytic inflammation (Fig. 8), and rarely formed small clusters within choroidal (pyo)granulomatous lesions.

Retinal lesions were identified in the same 20/24 cats (83%) that had lesions within the choroid, although the median inflammation score was lower (median score = 3). (Pyo)granulomatous inflammation was the dominant finding in 7 cats (cases 1, 2, 7–9, 14, and 17), with a mixed inflammatory population in 9 cats (cases 4–6, 10, 11, 13, 23, and 24) (Fig. 9). “Organized” granulomas were identified in 3 cats (cases 5, 13, and 18); otherwise, the inflammatory cell infiltrate was “unstructured.” The remaining 4 cases were dominated by lymphoplasmacytic inflammation (cases 12, 16, 19, and 20). Aggregates of perivascular lymphocytes were identified in 13 cats (cases 1, 4–7, 10, 13, 14, 16, 18, 19, 20, and 23); these cuffs comprised both T and B cells in variable numbers. Focal or complete retinal detachment, or evidence of antemortem retinal detachment, that is, hypertrophy of the retinal pigment epithelium (RPE) and/or subretinal inflammatory exudate, was present in 16/20 cats (80%) with retinal lesions (cases 1, 5–8, 10–14, 16–20, and 23). Within the subretinal space, a proteinaceous exudate, or proteinaceous debris, was identified in 15/16 cats (94%; cases 1, 5–8, 10–14, and 16–20); a concurrent cellular component was identified in 14/15 cats (93%; cases 1, 5–8, 10–14, 15, and 17–20), mostly consisting of Iba1- or calprotectin-positive cells, with fewer lymphocytes. Evidence of hemorrhage into the subretinal space, that is, erythrocytes and erythrophages, was seen in 8/16 cats (50%; cases 1, 8, 11, 13, 14, 18, 19, and 23). There was diffuse atrophy of the photoreceptor layer in all 20 cases; loss of the other outer retinal layers was variable and, in some cases, this extended to full-thickness retinal atrophy. Retinal necrosis was present in 15/20 cats (75%) and was typically diffuse or multifocal (cases 2, 4–11, 13, 14, 17–19, and 23). In cases of (pyo)granulomatous or mixed (pyo)granulomatous-lymphocytic retinitis, Iba1-positive cells were the most common cell population on IHC; some positive cells formed granulomas. There were positive cells on the inner limiting membrane of the retina, as well as scattered diffusely throughout multiple retinal layers (Fig. 10); some of these likely represented reactive and quiescent resident microglia, in addition to infiltrating macrophages. Small numbers of Iba1-positive cells were also identified in cases of lymphocytic retinitis. Calprotectin-positive cells were also present throughout the retina, but numbers of positive cells decreased toward the periphery of the retina. T cells were identified in all retinal lesions, often in association with areas of (pyo)granulomatous inflammation, but they were also diffusely scattered throughout the outer and inner retinal layers. As with choroidal lesions, B cells were less likely to be found in association with areas of (pyo)granulomatous inflammation; however, in lymphocyte-rich regions of retinitis, B cells were identified, sometimes in huge numbers.

### Anterior Uvea

**Figures 11–18.**
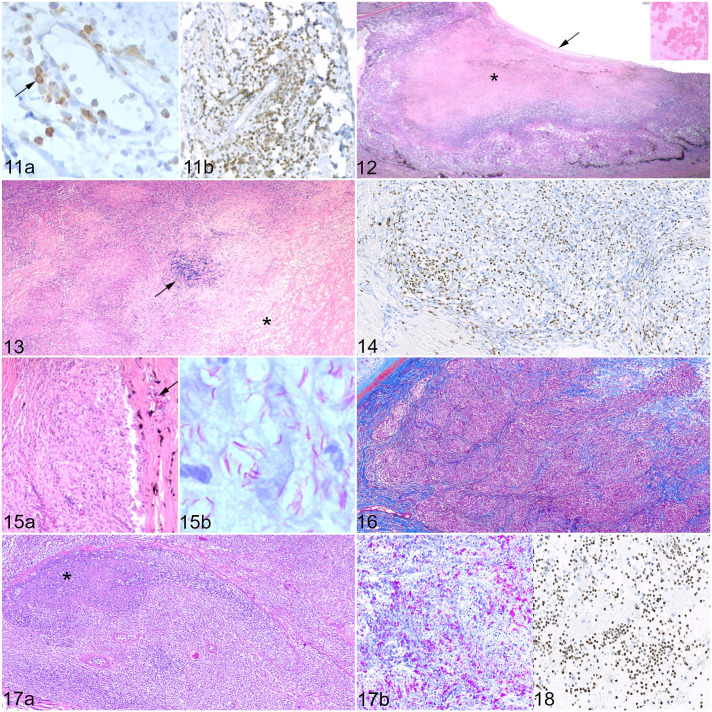
Mycobacteriosis, eye, cat. Inflammation scores: 1 = less than 1000 inflammatory cells; 2 = 1000–10,000 inflammatory cells; 3 = 10,001–50,000 inflammatory cells; 4 = 50,001–100,000 inflammatory cells; 5 = more than 100,000 inflammatory cells, calculated over the entire area of the lesion. **Figure 11.** Iris, *Mycobacterium bovis*, case 5. Inflammation score 2. (a) Lymphoplasmacytic iritis with scant T cells scattered throughout the iris stroma (black arrow). Immunohistochemistry (IHC) for CD3. (b) B cells are more abundant and outnumber T cells. IHC for Pax5. **Figure 12.** Ciliary body, *M. bovis*, case 9. Pyogranulomatous cyclitis, inflammation score 5, with extensive necrosis (asterisk) and loss of the epithelium of the pars plicata. There is a cyclitic membrane (black arrow), and Morgagnian globules in the lens (inset), indicating a cataract. Hematoxylin and eosin (HE). **Figure 13.** Sclera and episclera, *M. bovis*, case 4. Pyogranulomatous scleritis, inflammation score 5, with degeneration of collagen (asterisk) and a cluster of lymphocytes (black arrow). HE. **Figure 14.** Sclera, *Mycobacterium lepraemurium*, case 22. Pyogranulomatous scleritis, inflammation score 4, with “atypical” granulomas showing immunolabeled monocytes and granulocytes, mostly within the granuloma; macrophages and epithelioid macrophages are negative. IHC for calprotectin. **Figure 15.** Corneal limbus, *M. lepraemurium*, case 21. Inflammation score 3. (a) Pyogranulomatous perilimbal inflammation expanding the stroma, with the formation of non-necrotic pyogranulomas dominated by epithelioid macrophages. Normal features of the corneal limbus such as blood vessels (black arrow) and melanocytes are present. HE. (b) Positive staining for acid-fast bacilli indicative of mycobacteria within pyogranulomas. Bacterial index grade 5. Ziehl-Neelsen (ZN). **Figure 16.** Conjunctiva, *Mycobacterium microti*, case 3. Pyogranulomatous conjunctivitis of the third eyelid, inflammation score 5. Collagen fibers are present, subdividing the inflammatory infiltrate. The fibrous capsules are thin and incomplete. Masson’s trichrome. **Figure 17.** Optic nerve, *M. bovis*, case 8. Inflammation score 5. (a) Pyogranulomatous optic neuritis with necrosis (asterisk). HE. (b) Abundant acid-fast bacilli (pink) present within the optic nerve. The overall bacterial index grade was 5. ZN. **Figure 18.** Vitreous, *M. bovis*, case 17. Neutrophils are numerous within the vitreous. IHC for calprotectin.

Inflammatory cells were present in the iris of 16/24 cats (67%; cases 2, 4–7, 9–18, and 23). Typically, iridal lesions were characterized by lymphoplasmacytic inflammation with an inflammation score of 1 to 2 (11/16 cats, 69%; cases 2, 4, 5, 7, 10–14, 16, and 23); this was the only ocular structure where lymphoplasmacytic inflammation predominated. The remaining 5 cats showed (pyo)granulomatous inflammation or a mixed infiltrate (cases 6, 9, 15, 17, and 18). AFB were identified in 2 cats, both of which were characterized by pyogranulomatous inflammation; one scored as bacterial index grade 2 (low) (case 17) and the other as bacterial index grade 3 (high) (case 9). Lymphocytes and plasma cells often formed perivascular cuffs but were also identified throughout the iris stroma either forming loose aggregates of cells or were diffusely distributed; most cases showed greater numbers of B cells (Fig. 11a, b). In cases with (pyo)granulomatous or mixed inflammation, there was evidence of granuloma formation dominated by Iba1-positive macrophages, variable numbers of calprotectin-positive cells, and a peripheral layer of T cells. Concentrically arranged spindle-shaped cells were sometimes present, but there was no evidence of a fibrous capsule on staining with MT. Iba1-positive cells were infrequently identified lining the anterior iris epithelium. Extensive necrosis was present in 1 cat (case 17, inflammation score 5), resulting in the loss of the posterior pigmented iris epithelium. Pre-iridal membranes were identified in 20/24 cats (83%; cases 1, 2, 4–20, and 23), although in some cases these were very subtle, consisting of a focal, single-cell thickness cellular membrane on the anterior surface of the iris.

There was evidence of inflammation in the ciliary body in 20/24 cats (83%); all 16 cats with iridal inflammation demonstrated concurrent cyclitis (cases 2, 4–7, 9–18, and 23). Inflammation of the iris was not identified in the remaining 4 cats (cases 1, 8, and 20, inflammation score 1; case 19, inflammation score 2). Ten cats showed (pyo)granulomatous or mixed inflammation, with an inflammation score of 3 or greater. Granulomas were identified in 7 cats (“organized” cases 4, 9, 11, 16, and 17, “atypical” cases 2 and 12), whereas the remaining 3 cats showed diffuse “unstructured” infiltration of the ciliary body (cases 6, 7, and 18). In 2 cats with an inflammation score of 5 (cases 9 and 17), there was extensive destruction and necrosis of the pars plicata (Fig. 12).

In 14/24 cats (58%), there was extension of inflammatory cells from the base of the iris or the ciliary body into the trabecular meshwork (cases 2, 5–7, 9–12, 14–18, and 23), and in 3 cats there was collapse of the iridocorneal drainage angle (cases 6, 16, and 17). In 10 cats, this inflammatory cell population was predominated by Iba1- or calprotectin-positive cells, with variable numbers of T and B cells (cases 5, 6, 9, 11, 12, 14, 16–18, and 23). Lymphoplasmacytic infiltration of the trabecular meshwork dominated in 4 cats, of which 3 showed greater numbers of T cells (cases 2, 7, and 15), whereas B cells dominated in the remaining cat (case 10). The optic disc was not present in the examined sections to evaluate for cupping, which would indicate glaucoma. However, there was clinical suspicion of glaucoma reported in the history provided for 3 cases (cases 6, 17, and 18).

### Cornea, Sclera, and Conjunctiva

Inflammatory cells were present within the sclera in 20/24 cats (83%; cases 1, 2, 4–10, 12–14, 16–22, and 24), with a median inflammation score of 2, although an inflammation score of 5 was recorded in 4 cats (cases 4, 9, 12, and 16). Perivascular mixed inflammation (inflammation scores 1–2) was the sole scleral change identified in 8 cats (cases 5, 6, 10, 13, 14, 17, 20, and 24), with a further 2 cats showing perivascular inflammation in conjunction with other inflammatory lesions within the sclera (cases 2 and 18). Extension of (pyo)granulomatous choroidal lesions into the posterior sclera was recorded in 4 cats (cases 1, 7, 8, and 19), a nodular (pyo)granulomatous anterior scleritis was present in 4 cats (cases 2, 16, 21, and 22), and (pyo)granulomatous scleritis as part of an orbital or periorbital cellulitis was identified in 3 cats (cases 4, 9, and 12). Granulomas or pyogranulomas were present in 9 cats with (pyo)granulomatous inflammation (cases 2, 4, 9, 12, 16, 18, 19, 21, and 22), compared with 3 cats with an “unstructured” inflammatory infiltrate (cases 1, 7, and 8). Where (pyo)granulomas were present, they were classified as “organized” in 4 cats (cases 4, 9, 12, and 16); fibrous encapsulation of (pyo)granulomas ranged from absent to thick, well-formed capsules. Scleral collagenolysis was identified in cats with an inflammation score of 4 or 5 (Fig. 13). As with lesions in other tissues, Iba1-positive macrophages were the most common cell type on IHC, followed by calprotectin-positive cells (Fig. 14), and with lesser numbers of T and B cells, and a similar pattern of distribution.

Corneal lesions were identified in 6/24 cats (25%; cases 2, 9, 16, 18, 21, and 22), with a median inflammation score of 2.5. Five of the lesions were (pyo)granulomatous, dominated by Iba1-positive epithelioid macrophages; 4 of these formed “atypical” (pyo)granulomas with varying degrees of encapsulating fibrosis (cases 2, 9, 21, and 22), whereas case 16 showed “organized” (pyo)granulomas. Corneal lesions were contiguous with a lesion that extended into the sclera, bulbar conjunctiva, and episcleral tissues (cases 2, 16, and 22). In case 21, the corneal lesion was contained within the stroma (Fig. 15a, b). Corneal vascularization was identified in all 6 cats.

Infiltration of the conjunctiva with inflammatory cells was recorded in 10/24 cats (42%; cases 2–4, 9, 10, 12, 13, 16, 22, and 23), with a median inflammation score of 2. Two cats had an inflammation score of 5 (cases 3 and 9), both of which consisted of pyogranulomatous inflammation dominated by Iba1-positive macrophages and epithelioid macrophages and had a high bacterial index grade (grades 4 and 5, respectively). In 1 cat, the inflammatory cell population was “unstructured,” with mixed regions of fibrosis, necrosis, and edema, as part of a periorbital cellulitis (case 9); case 3 consisted of a large confluent sheet of “atypical” pyogranulomas diffusely expanding the nictitating conjunctiva. Extensive regions of fibrosis, with areas of necrosis, were also present (Fig. 16). “Atypical” granulomas with encapsulating fibrosis and bridging populations of T cells were also identified in the second cat with *M. microti* infection (case 2); the conjunctiva was involved as part of a corneal-scleral-conjunctival mass. Another cat with a contiguous corneal-scleral-conjunctival mass consisted of “organized” pyogranulomas, dominated by Iba1-positive macrophages and calprotectin-positive cells, with variable degrees of fibrosis and some regions of necrosis (case 16). The remaining 5 cats showed diffuse infiltration with small numbers of calprotectin-positive cells, T cells, and fewer B cells.

### Optic Nerve

Inflammation of the optic nerve was present in 11/20 cats (55%; cases 1, 6–9, 12–14, 20, 23, and 24), with a median inflammation score of 2; the optic nerve was not present for histopathological assessment in 4 cats (cases 4, 5, 21, and 22). Pyogranulomatous or mixed inflammation, dominated by Iba1-positive macrophages and epithelioid macrophages, was present in 9 cats (cases 1, 6–8, 12–14, 20, and 24), compared with 2 cats (cases 9 and 23) where B cells were the predominant cell type. In both cats, lymphocytes were found to accumulate around rather than directly infiltrate the optic nerve. Two cats had an inflammation score of 5. In one (case 12), small, mostly “atypical” (pyo)granulomas were present, expanding the optic nerve; however, AFB were not detected in this tissue. Extensive necrosis and infiltration of the optic nerve with “unstructured” pyogranulomatous inflammation with minimal fibrosis was identified in the second cat (case 8); AFB were numerous (bacterial index grade 5), especially within regions of necrosis (Fig. 17a, b). In the remaining cats, there was infiltration of the optic nerve head and pial trabeculae with pyogranulomatous inflammation and a lesser lymphocytic component.

### Anterior Chamber, Lens, and Vitreous Chamber

A proteinaceous effusion, or proteinaceous debris such as fibrin clots, was identified in the anterior chamber in 9 cats (cases 6, 7, 9, 11, 13, 14, 17, 18, and 23). In these cases, plus an additional 3 (cases 2, 8, and 16), inflammatory cells were present, some of which appeared adherent to the corneal endothelium. These cells were mostly calprotectin-positive, with fewer T cells and B cells.

A cataract was identified in 5 cats (cases 2, 4, 9, 17, and 23), and posterior synechiae were present in 3 cats (cases 6, 17, and 18). Rupture of the posterior lens capsule with neutrophilic phakitis and intralenticular AFB was identified in 1 cat (case 17).

Predominantly calprotectin-positive inflammatory cells with fewer macrophages and lymphocytes were present in appreciable numbers within the vitreous in 3 cats (cases 8, 17, and 18; Fig. 18). Free AFB were present within the vitreous of the single case with posterior lens capsule rupture and phakitis (case 17).

## Discussion

This study enabled detailed description of the histopathological and immunohistochemical features of feline ocular mycobacterial lesions. The most affected ocular tissues were the choroid, retina, ciliary body, and sclera, with the greatest degree of inflammation present within the choroid. In all cases where there was choroidal inflammation, there was concurrent involvement of the retina (ie, chorioretinitis). AFB were present in 20/24 cats (83%) and were most frequently found in choroidal lesions. Iba1-positive macrophages were the most common population based on IHC, followed by calprotectin-positive granulocytes and monocytes, T cells, and then B cells, although this did vary between the affected tissue, both between and within individual cats. A diagnosis of TB was made in 20/22 cats (91%) that underwent culture, PCR, or IGRA; the remaining 2 cats were diagnosed to have *M. lepraemurium*, which has recently been identified as a cause of ocular disease in the cat.^30^

Reports of feline ocular mycobacteriosis typically describe clinical signs attributable to uveitis, as well as loss of vision.^82^ While inflammatory lesions were identified across all tissue types examined, the most consistent histopathological finding was posterior or panuveitis with concurrent retinitis, which was identified in 20/24 cases (83%) in this study, a higher proportion compared with what has been previously reported.^82^ In this study, the median inflammation score was greatest for choroidal lesions, followed by the retina. Posterior or panuveitis is the most common histopathological presentation of cases of ocular TB in adults and children,^90^ suggesting similarities between the underlying pathogenesis of feline and human ocular mycobacteriosis. Choroidal lesions have also been produced in guinea pigs experimentally infected with aerosolized *M. tuberculosis*.^70,86^ The distribution of ocular mycobacterial lesions predominantly locating to the choroid resembles cases of ocular metastatic neoplasia in cats, where lesions are typically found in the choroid rather than the anterior uvea, in contrast to the distribution of metastatic neoplastic lesions in dogs.^21^ Given these commonalities between the distribution of lesions in cases of feline ocular metastatic neoplasia and ocular mycobacterial lesions, it provides some strength to the hypothesis that ocular mycobacterial lesions arise following hematogenous dissemination of mycobacteria to the eye. The choroid is a highly vascular structure supplying oxygen and nutrition to the retina.^58^ Choroidal lesions can result in ischemia,^86^ with subsequent atrophy of the outer retinal layers.^20^ Ischemia of the choriocapillaris and RPE can result in breakdown of the blood-retinal barrier and the development of a subretinal exudate, leading to retinal detachment.^52^ This results in rapid morphological changes in the feline retina, including proliferation of the RPE and photoreceptor atrophy.^5,20^ Where chorioretinitis was present, concurrent retinal detachment was identified in most, but not all, cases in this study. In addition, retinal necrosis and atrophy were consistent findings, in particular atrophy of the photoreceptor layer which was present in all cats with retinal inflammatory lesions. The RPE plays a role in limiting ocular mycobacterial infection and can control growth of mycobacteria,^57^ as well as serve to reduce ocular inflammation via interferon-signaling pathways.^38,44^ This may in part explain the lower median inflammation score for retinal lesions compared with those in the choroid, but there is a delicate balance to strike between reducing T cell recruitment to regulate the inflammatory response, while providing adequate control of infection. The overwhelming inflammatory changes present within the choroid, resulting in damage to the choriocapillaris and RPE, may therefore result in loss of this control mechanism, resulting in cases with more severe retinal lesions.

Inflammation of the optic nerve and/or sclera in conjunction with posterior or panuveitis and retinitis was common. Infiltration of the optic nerve and its surrounding sheath could result from extension of choroidal lesions, as inflammatory cells were typically concentrated around the head and intrascleral component of the optic nerve. Mycobacterial optic neuritis is infrequently recognized in humans,^66,90^ and it may represent a retrobulbar complication of meningitis.^36^ These findings suggest optic nerve involvement in feline ocular mycobacteriosis may be under-recognized. Scleral involvement was either limited to perivascular aggregates of inflammatory cells, as part of an anterior mass contiguous with corneal and/or conjunctival involvement, extension of choroidal lesions, or as part of an overwhelming (peri)orbital disease process, all of which have been recorded in human ocular mycobacteriosis.^69,79^ Conjunctival and corneal granulomas were less frequently identified, which is consistent with reports of ocular mycobacteriosis in humans.^90^ Three cats in this study showed inflammatory changes restricted to the outer coat of the eye (cornea, sclera, and conjunctiva). This could result from direct inoculation of mycobacteria or contamination of a wound following an ocular injury,^4^ or it could represent a delayed-type hypersensitivity reaction to remote mycobacterial antigens.^4,68^ The exact mechanism by which remote mycobacterial antigens elicit a type IV hypersensitivity response in the outer tunic of the eye is not fully understood.^66^ The cornea, sclera, and/or conjunctiva may become sensitized to mycobacterial antigens from the environment. Then, when these tissues are “re-introduced” to circulating antigens in the bloodstream or in ocular secretions in cases of mycobacterial infection at a distant site in the body, lesions may subsequently develop.^22^ Alternatively, these lesions may arise from direct exogenous inoculation. Finally, anterior uveitis without evidence of choroidal involvement was identified in 1 cat. This has been recorded in cats and humans^82,90^ but appears to be uncommon. Iridal (pyo)granulomas were characterized by a lack of a fibrous capsule. The fibrous capsule associated with mycobacterial (pyo)granulomas consists of type I collagen.^88,89^ It has been shown that in cases of ocular inflammation in nonhuman primates, levels of type I collagen in the extracellular matrix can be reduced, presumably due to the increased concentration of prostaglandins within the aqueous humor.^76^ The lack of anterior iris epithelium allows for fluid exchange across the iris and the anterior chamber, and in the case of ocular inflammation, this increase in permeability may allow for increased exchange and activity of antifibrotic prostaglandins in the aqueous humor on resident iris fibroblast populations, inhibiting collagen synthesis and secretion to form a capsule around (pyo)granulomas. The uveal tract is highly resistant to fibrosis, as the formation of scar tissue could be detrimental to the functioning of the eye^21^; interestingly, choroidal fibrosis was identified in 60% of cases in this study. Fibrosis within the choroid is associated with choroidal rupture, often following direct or indirect trauma to the globe, including penetrating injuries,^3^ which could also act as a route of transmission of mycobacteria into the eye. However, histopathological evidence of trauma to the globe was not identified in these cases. These findings would suggest the choroid is the primary site of lesion development in most cases of feline ocular mycobacteriosis, with subsequent extension to the retina and then the rest of the uveal tract and other ocular tissues, depending on the balance of ocular inflammatory mechanisms. However, there is case-by-case variation presumptively in association with the route of infection.

The typical histopathological appearance of feline mycobacterial lesions is that of granulomatous to pyogranulomatous inflammation, dominated by epithelioid macrophages, and a relative lack of multinucleated giant cells.^35,41,55^ In this study, (pyo)granulomatous inflammation was the dominant reaction in ocular lesions, other than those in the iris, where lymphoplasmacytic inflammation was more common. Mixed (pyo)granulomatous-lymphocytic inflammation was frequently documented, especially in the choroid and ciliary body. Typically, this consisted of an accumulation of epithelioid macrophages, monocytes, neutrophils, and fewer lymphocytes within the inner layers of the choroid, with aggregates of lymphocytes and plasma cells around the larger vessels in the outer choroid. Segmental lymphoplasmacytic infiltration within regions of (pyo)granulomatous inflammation in the choroid was also documented. Previous reports on the histopathology of feline ocular mycobacteriosis lesions have not described significant involvement of lymphocytes,^82^ although infiltration with lymphocytes in the outer choroid has been documented in humans.^9^ Where there is a predominance of lymphocytic infiltration of ocular tissues in cases of mycobacteriosis this may reflect a nonspecific immune response,^12^ it could suggest differences in ocular responses to mycobacteria due to its immunoprivileged nature,^83^ or it may reflect the chronicity of the lesion. Alternatively, the lymphoplasmacytic anterior uveitis may be unrelated to the mycobacterial infection and simply reflect a concurrent inflammatory process occurring within the eye.^51^ A recent histopathological study into cases of FIP with ocular lesions demonstrated that B cells and plasma cells were the most common populations present in cases of severe and extensive inflammation, and that macrophages were not the predominant inflammatory cell in contrast to FIP lesions in other organs,^91^ suggesting there may be unique immunological features pertaining to the eye. Feline tuberculous granulomas have been recently categorized as “organized” or “atypical,” depending on the features exhibited in these lesions.^55^ These granulomas were infrequently identified across all ocular tissues, but many lesions exhibited an “unstructured” inflammatory infiltrate. Compared with other species, “classical” granuloma formation, that is, central necrosis and an extensive fibrous capsule,^88^ is not a routine feature of mycobacterial lesions in cats,^35,41^ which may reflect differences in the feline immune response or the inciting pathogen, with cases of *M. microti* more likely to present with “atypical” granulomas than “organized” granulomas.^55^ Despite this, the identification of (pyo)granulomatous inflammation as the main histopathological presentation of feline ocular mycobacterial lesions suggests a broadly conserved immunological response to these infections in cats, regardless of the location of lesions.

This study identified AFB morphologically consistent with mycobacteria in 83% of cases, a substantially higher number than detected in previous studies.^33^ This could result from selection bias of the cases included in this study, but it has been demonstrated that detection of AFB is dependent on the experience of the individual reading the slide.^35^ Detection of AFB in human ocular mycobacterial samples is uncommon, and if identified organisms are typically rare.^90^ While mycobacteria can change the composition of their cell wall, resulting in reduced sensitivity with ZN staining,^78^ this alone is unlikely to explain the difference between the abundance of ZN-positive cases in this study compared with what has been identified in human studies. Therefore, there may be differences in the pathogenesis of these infections. Given the lack of identification of mycobacteria in human ocular samples, either through ZN staining or molecular methods,^1,37,90^ it has been proposed that some cases of human ocular TB represent a systemic inflammatory response or result from antigenic mimicry between mycobacterial and retinal antigens,^6,9,29,87^ rather than (peri)ocular infection. In this study, AFB were detected in 16/20 choroidal lesions (80%), with over half classified with a high bacterial index grade. A bacterial index grade of 5 (100–1000 AFB/high-power field) was also recorded for lesions in the cornea, sclera, conjunctiva, retina, and optic nerve. In 1 cat, AFB were detected within the lens and vitreous. The presence of AFB within a range of tissues suggests the inflammatory changes present within the eye are driven by active infection, rather than a systemic inflammatory response or antigenic mimicry, hence topical anti-inflammatory therapy alone would not be advisable. An additional finding was that high bacterial index grades were identified in cases of infection with both *M. bovis* and *M. microti*, as well as 1 case of *M. lepraemurium* infection, indicating that the presence of numerous AFB is not restricted to nontuberculous mycobacterial infections.^48^

Immunohistochemical analysis of mycobacterial lesions has been performed on many species,^27,28,88^ including cats,^41,55^ and can be a useful tool to describe the cellular populations present and infer the underlying immunological processes taking place. Abundant positive labeling for Iba1 was identified in ocular mycobacterial lesions in this study, confirming the presence of macrophages. Macrophages are the hallmark cell of mycobacterial infections, playing key roles both promoting and modulating the immune response through the secretion of pro- and anti-inflammatory cytokines and chemokines, as well as phagocytosing bacteria,^61^ but they can also be manipulated by mycobacteria to promote their own intracellular survival, as well as allow for trafficking from the primary site of infection to other parts of the body.^26^ Retinal and optic nerve microglia were also identified; Iba1-positive cells were more abundant in cats with retinitis and optic neuritis, respectively, compared with cats where inflammatory changes were not associated with these tissues. Iba1 expression is upregulated by reactive microglia,^47^ which can play an active role in presenting antigens to CD4+ T cells, as well as driving further recruitment of immune cells.^81^ Calprotectin-positive cells were abundant in regions of (pyo)granulomatous inflammation, suggesting recent recruitment of circulating monocytes and neutrophils, and active development of ocular lesions.^74^ As expected, epithelioid macrophages did not express this molecule. A zebrafish model of ocular mycobacteriosis identified peripheral blood monocytes in developing granulomas within the choroid-RPE complex in the face of a grossly intact blood-retinal barrier, whereas these cells were not identified in uninfected eyes, suggesting breach of the normal mechanisms that confer ocular immune privilege.^85^ Our findings suggest that monocyte-recruitment signaling pathways are preserved in cases of ocular mycobacteriosis,^17^ with subsequent maturation of peripheral monocytes to Iba1-positive, calprotectin-negative epithelioid macrophages. Lymphocytes were detected using the T and B cell-specific molecules CD3 and Pax5, respectively. While the proportion of cells expressing these markers varied between cases and tissue, T cells were more commonly identified than B cells, which is consistent with what has been previously reported in feline mycobacterial lesions.^41,55^ Positive labeling for CD3 showed T cells were present within regions of both (pyo)granulomatous and lymphocytic inflammation, whereas B cells were mostly restricted to regions of lymphoplasmacytic inflammation. The role of T cells in mycobacterial infections has been well described,^61^ whereas B cells may regulate T cell responses and drive modulatory inflammatory responses.^53^ Subsets of T cells, including proinflammatory T helper 17 and T regulatory cells, may also be present. The role of these cells has been explored in mycobacterial infections,^11,50,77,80^ and a small study has identified T helper 17 cell involvement in feline idiopathic anterior uveitis, but not in cases of FIP-induced anterior uveitis.^12^ Exploring these cell populations may provide further insight into the underlying immunological mechanisms controlling feline ocular mycobacterial infections. Autoreactive T cells have also been identified in samples from cases of human ocular TB^84^; whether they play a contributory role in the pathogenesis of feline ocular mycobacteriosis has yet to be elucidated.

While histopathology may be sufficient to obtain a diagnosis of mycobacterial infection, further testing via specialized culture, PCR, or IGRA is required to identify, or infer, infection with a specific species or group of mycobacteria. In this study, 20/22 cats (91%) that underwent either culture, PCR, or IGRA were diagnosed with TB. A diagnosis of *M. bovis* infection was made in 11/20 cats (55%), compared with 2/20 (10%) with *M. microti*; the species could not be established for the remaining 7/20 (35%). Of these 20 cats with TB, posterior or panuveitis and retinitis with or without optic neuritis or scleritis was reported in 18; the remaining 2 cats presented with histologic lesions of anterior uveitis (MTBC) or conjunctivitis (*M. microti*). Historically, cases of retinal detachment in cats were associated with *M. bovis* infection,^23^ and the findings of this study would suggest that most cases of intraocular mycobacterial disease are due to *M. bovis*. It has been suggested that the virulence factors of early secretory antigenic target 6 kDa, culture filtrate protein 10 kDa, and heparin-binding hemagglutinin adhesin play an important role of *M. tuberculosis* translocation from the lung to extrapulmonary sites.^17,43,75^ These virulence factors are also encoded by *M. bovis*, but either deleted or present in only some strains of *M. microti*,^24,65^ and therefore this pathogen may show reduced capacity to reach organs such as the eye following primary inoculation and infection at another site. Two cats were diagnosed with *M. lepraemurium* infection, which typically causes cutaneous lesions, sparing the eye.^60^ In this study, cases of *M. lepraemurium* infection were restricted to the cornea, sclera, and conjunctiva. The external location of these lesions would suggest local infection of the ocular or periocular tissues rather than dissemination from a distant site. Naturally occurring infections with *M. lepraemurium* across species are restricted to the external tissues, which may be in part due to inherent qualities of the bacterium. For example, *M. lepraemurium* may not be able to replicate as well at higher body temperatures compared with the cooler body surface tissues,^13^ hence why these lesions were restricted to the external tunic of the eye. It is therefore important to recognize that *M. bovis* appears to be the most common cause of feline ocular mycobacteriosis in the United Kingdom and should be a differential diagnosis in cases of retinal detachment, while other species of mycobacteria, including *M. lepraemurium*, can cause ocular disease in the cat.

There has been renewed interest in animal models of ocular TB,^10^ and it has been suggested that cats may present an alternative naturally occurring model.^56^ The cat has previously been suggested as a model for human ocular toxoplasmosis,^15^ although it has become apparent that there are differences in the pathogenesis of ocular toxoplasmosis between cats and humans.^14^ Similarly, the data from this study suggest some similarities, but also differences, in the distribution of ocular mycobacterial lesions, the histopathological changes present, and the presence of AFB. Further descriptions of the clinical presentations of cases of feline mycobacteriosis are needed, with a particular focus on retinal examination, even in cases where ocular disease is not clinically apparent. This may identify ocular involvement at an early stage with different phenotypic presentations of disease.

In summary, this study has shown that ocular lesions associated with feline mycobacterial infection can be found in all tissues and typically consisted of granulomatous to pyogranulomatous inflammation, with a substantial lymphocytic component identified in many tissues, in particular the iris. The choroid, retina, ciliary body, and sclera were the most frequently affected tissues, with the highest median inflammation score associated with choroidal lesions. AFB were identified in nearly all cases, especially within the choroid, suggesting hematogenous dissemination of mycobacteria to the eye. While the most frequent histological finding was chorioretinitis, cases presenting without posterior segment or intraocular disease were also identified; these were thought to arise from inoculation of the eye following traumatic injury. Nearly all cases of ocular mycobacteriosis were diagnosed as TB, and where speciation was available, *M. bovis* was identified more frequently than *M. microti*. This study also demonstrated 2 cases of ocular *M. lepraemurium* infection. In conclusion, the cat may provide some insight into human cases of ocular mycobacteriosis.

## Supplemental Material

sj-pdf-1-vet-10.1177_03009858221098431 – Supplemental material for Ocular mycobacterial lesions in cats Supplemental material, sj-pdf-1-vet-10.1177_03009858221098431 for Ocular mycobacterial lesions in cats by Jordan L. Mitchell, Laura MacDougall, Melanie J. Dobromylskyj, Ken Smith, Renata Stavinohova, Danièlle A. Gunn-Moore, Jayne C. Hope and Emma Scurrell in Veterinary Pathology
