# Supplementary material for: Ocular mycobacterial lesions in cats
Source: Vet Pathol. 2022 May 19;59(5):792–805. doi: 10.1177/03009858221098431 (PMC9358306; doi:10.1177/03009858221098431)
Supplement: sj-pdf-1-vet-10.1177_03009858221098431 – Supplemental material for Ocular mycobacterial lesions in cats [file sj-pdf-1-vet-10.1177_03009858221098431.pdf]

Supplemental Table S1: Summary of case details for cats involved in this study.

Case No.	Age	Gender	Breed	Culture	PCR	IGRA	FeLV antigen	FIV antibody	Stavinohova *et al.** Case No.
1	6y	MN	Bengal	*M. bovis*	Negative	*M. bovis*	Negative	Negative	10
2	8y	FN	DSH	*M. microti*	NP	MTBC	NP	NP	6
3	11y	MN	DSH	*M. microti*	NP	MTBC	NP	NP	-
4	10y	MN	BSH	NP	*M. bovis*	NP	NA	NA	20
5	4y	F	DxH	NP	*M. bovis*	NP	NA	NA	-
6	8y	MN	DSH	NP	*M. bovis*	NP	NA	NA	21
7	7y	F	DxH	NP	*M. bovis*	NP	NA	NA	-
8	1y	M	NA	NP	*M. bovis*	NP	NA	NA	-
9	5y 4m	FN	DSH	NP	*M. bovis*	NP	NA	NA	-
10	10y 5m	MN	DSH	NP	MTBC	*M. bovis*	Negative	Negative	-
11	3y	FN	DSH	NP	MTBC	MTBC	NP	NP	-
12	7y	MN	DSH	NP	MTBC	MTBC	NP	NP	-
13	4y	MN	DSH	NP	MTBC	NP	NA	NA	14
14	2y	MN	DSH	NP	MTBC	NP	NA	NA	17
15	6y	MN	BSH	NP	MTBC	NP	NA	NA	18
16	11y	M	DSH	NP	MTBC	NP	NA	NA	-
17	7y 2m	MN	Burmese	NP	NP	*M. bovis*	Negative	Negative	3
18	1y 9m	MN	DSH	Negative	Negative	*M. bovis*	NP	NP	2
19	2y 1m	FN	DSH	NP	NP	*M. bovis*	Negative	Negative	-
20	2y	FN	BSH	NP	NP	MTBC	NP	NP	-
21	12y 6m	FN	DLH	NP	*M. lepraemurium*	NP	NA	NA	-
22	10y	F	DxH	NP	*M. lepraemurium*	NP	NA	NA	-
23	3y	FN	DLH	NP	NP	NP	NP	NP	-
24	1y 6m	FN	DSH	NP	NP	NP	NP	NP	22

PCR = polymerase chain reaction, IGRA = interferon-gamma release assay, FeLV = feline leukaemia virus, FIV = feline immunodeficiency virus, y = years, m = months, M = male, F = female, N = neutered, DSH = domestic short-hair, DxH = domestic cat, DLH = domestic long-hair, BSH = British Shorthair, MTBC = *Mycobacterium tuberculosis*-complex, NP = not performed, NA = data not available

*Stavinohova R, O'Halloran C, Newton JR, Oliver JAC, Scurrell E, Gunn-Moore, DA. Feline Ocular Mycobacteriosis: Clinical Presentation, Histopathologic Features, and Outcome. *Veterinary Pathology*. 2019;56: 749-760.

Supplemental Table S2: Breakdown of inflammation score, inflammation type and bacterial index grade per tissue examined per case.

Case No.	Cornea			Conjunctiva			Sclera			Iris			Ciliary Body			Choroid			Retina			Optic Nerve		
	IS	IT	BI	IS	IT	BI	IS	IT	BI	IS	IT	BI	IS	IT	BI	IS	IT	BI	IS	IT	BI	IS	IT	BI
1	-	-	-	-	-	-	3	PG	4	-	-	-	1	L	0	5	PG	4	2	PG	0	3	M	0
2	5	PG	3	3	M	2	3	M	4	2	L	0	4	M	3	4	M	4	2	PG	0	-	-	-
3	-	-	-	5	PG	4	-	-	-	-	-	-	-	-	-	-	-	-	-	-	-	-	-	-
4	-	-	-	2	M	0	5	PG	2	2	L	0	3	M	0	5	M	3	2	M	3	NP	-	-
5	-	-	-	-	-	-	1	M	0	2	L	0	2	M	0	4	M	3	4	M	2	NP	-	-
6	-	-	-	-	-	-	1	M	0	2	M	0	3	PG	4	3	PG	4	2	M	4	3	PG	3
7	-	-	-	-	-	-	3	PG	0	2	L	0	3	M	0	5	PG	5	3	PG	4	3	PG	0
8	-	-	-	-	-	-	2	PG	2	-	-	-	1	PG	0	5	PG	5	3	PG	5	5	PG	5
9	2	PG	0	5	PG	5	5	PG	4	3	M	3	5	PG	4	5	PG	4	3	PG	3	2	L	0
10	-	-	-	1	L	0	1	M	0	1	L	0	2	L	0	5	M	4	3	M	3	-	-	-
11	-	-	-	-	-	-	-	-	-	2	L	0	3	PG	3	4	M	3	4	M	0	-	-	-
12	-	-	-	2	M	0	5	PG	0	1	L	0	5	PG	1	5	M	1	2	L	0	5	PG	0
13	-	-	-	1	M	0	1	M	0	1	L	0	1	L	0	5	PG	3	4	M	0	2	PG	0
14	-	-	-	-	-	-	2	M	0	1	L	0	2	M	0	3	M	2	4	PG	0	2	M	0
15	-	-	-	-	-	-	-	-	-	3	PG	0	2	L	0	-	-	-	-	-	-	-	-	-
16	4	PG	0	4	PG	0	5	PG	3	2	L	0	3	PG	0	3	PG	0	2	L	0	-	-	-
17	-	-	-	-	-	-	2	M	0	5	PG	2	5	PG	4	5	M	2	4	PG	3	-	-	-
18	1	L	0	-	-	-	2	M	0	2	M	0	4	M	0	3	PG	0	3	M	2	-	-	-
19	-	-	-	-	-	-	3	PG	0	-	-	-	2	PG	0	5	PG	1	3	L	0	-	-	-
20	-	-	-	-	-	-	2	M	0	-	-	-	1	L	0	5	M	1	3	L	0	2	M	0
21	3	PG	5	-	-	-	2	PG	5	-	-	-	-	-	-	-	-	-	-	-	-	NP	-	-
22	2	PG	0	2	PG	0	4	PG	0	-	-	-	-	-	-	-	-	-	-	-	-	NP	-	-
23	-	-	-	2	M	0	-	-	-	2	L	0	2	M	0	3	M	0	3	M	0	2	L	0
24	-	-	-	-	-	-	2	M	0	-	-	-	-	-	-	5	M	0	3	M	0	2	M	0

IS = inflammation score. IT = inflammation type. BI = bacterial index grade. PG = (pyo)granulomatous. M = mixed (pyo)granulomatous and lymphoplasmacytic inflammation. L = lymphoplasmacytic inflammation. NP = not present. - = not graded due to no inflammation present
